# The Significance of Nanomineral Particles during the Growth Process of Polymetallic Nodules in the Western Pacific Ocean

**DOI:** 10.3390/ijerph192113972

**Published:** 2022-10-27

**Authors:** Qiangtai Huang, Bo He, Zhourong Cai, Qianru Huang

**Affiliations:** 1 School of Marine Sciences, Sun Yat-sen University, Zhuhai 519082, China; 2 Guangdong Provincial Key Laboratory of Marine Resources and Coastal Engineering, Zhuhai 519082, China

**Keywords:** polymetallic nodules, nanomineral particles, growth process, Western Pacific Ocean

## Abstract

As a huge reservoir of economic metallic elements, oceanic polymetallic nodules have important strategic significance and are one of the main research objects in marine geology, especially their formation process and genetic mechanism. In this study, polymetallic nodules from the cobalt-rich crust exploration contract area in the Western Pacific Ocean were taken as the research object. Optical microscopy, scanning electron microscopy (SEM), X-ray diffraction (XRD), and energy dispersive spectroscopy (EDS) were used for observation and testing. The results indicate that many nanomineral particles, mainly composed of Fe and Mn, developed in polymetallic nodules from the western Pacific Ocean. The solid–liquid interface process of nanomineral particles plays an important role in the growth and evolution of nodules. We propose that the growth process of polymetallic nodules in the western Pacific Ocean can be divided into three stages. First, terrigenous detritus nucleates, and nanomineral particles composed of Fe, Mn, and other elements form, aggregate and attach to the core to form the initial shell. Second, a dense layer of the shell forms under stable conditions. In the third stage, the redox conditions of the nodules change, and the polymetallic nodules experience a variety of interface process modifications.

## 1. Introduction

Metal resources are of important economic value and strategic position in human society. With continuous development and utilization, the reserves of terrestrial metal resources are increasingly exhausted. Polymetallic nodules are widely distributed in the shallow layer or surface of sediments in deep-sea sedimentary basins ranging from 4000 to 6500 m in water depth, and they are composed of cores and shells rich in Mn, Ni, Cu, Co and REY metal elements; their reserves can reach tens or even thousands of times to those of terrestrial resources [1,2,3,4]. As one of the most important deep-sea mineral resources, the commercial exploitation feasibility of polymetallic nodules has attracted large-scale research, exploration and development worldwide [5]. The Pacific Ocean has the most abundant deposits, accounting for approximately 40% of the global distribution area of polymetallic nodules [6,7].

Polymetallic nodules are usually dark brown to black globular or elliptical masses several centimetres in diameter. The core consists of a variety of components, including fragments of earlier secondary nodules, altered basalt, hard parts of glass, clay, tuffaceous material, or living things [8,9]. The shell around the core is mainly composed of ferro-manganese minerals with a low degree of crystallization, showing a concentric ring around the core.

The different types of nodules usually form in different geochemical environments and have corresponding mineral compositions and geochemical characteristics. There are different viewpoints on the origin of polymetallic nodules; the most widely accepted genetic types are divided into hydrogenetic, diagenetic and hydrothermal origins according to the composition of polymetallic nodules [10,11,12,13].

Nanomaterial particle refers to a mineral in which the crystal size is smaller than 100 nm on a one-dimensional scale [14]. Nanomineral particles are abundant and widely distributed in nature. Due to their small size, most particles comprising atoms are on the particle surface or near the surface of nanomineral particles which have significantly different structures, physicochemical properties and activities compared with macroscopic substances [15,16,17,18]. Aggregation research is well developed, but aggregation research in the field of nanomaterials is a serious challenge due to unique physical and chemical properties [19]. Compared with ordinary minerals in nature, nanomineral particles have smaller particle sizes, larger specific surface areas and relatively larger surface energies; therefore, homogeneous aggregation is more likely to occur and form larger aggregates [17,20].

As nanomineral particles, ferro-manganese minerals in nodules are more likely to aggregate and grow in water, forming larger particles and promoting the growth of nodules. The aggregation behaviour of nanoparticles that influence the growth of oceanic polymetallic nodules is controlled by environmental factors (pH, ionic strength, and type and concentration of natural organic matter) and their own properties (particle size and morphology) [21,22,23].

As the main constituent minerals of deep-sea polymetallic nodules, ferro-manganese oxides and hydroxides are typical nanomineral particles. In this study, the genesis of polymetallic nodules was analysed by studying the microstructure, mineral composition and geochemical characteristics of polymetallic nodules. The typical nanomineral particles in nodule samples and their effects on nodule growth are discussed. These research achievements have important significance for further understanding of the formation process and genetic mechanism of polymetallic nodules.

## 2. Sampling Setting

**Figure 1:**
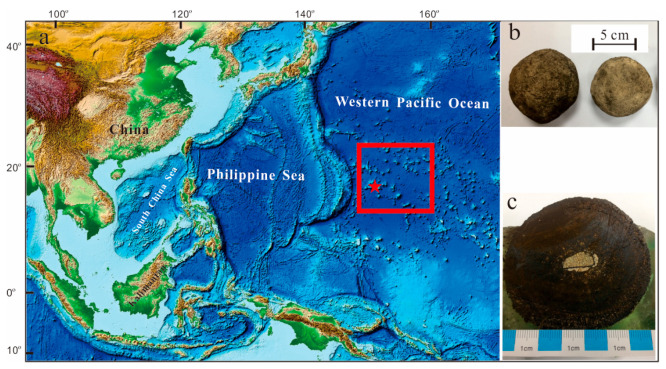
Sampling location map (**a**) and samples of polymetallic nodules (**b**,**c**). The red box indicates the cobalt-rich crust exploration contract area, and the asterisk indicates the sampling point.

The polymetallic nodules used in this study were collected from the cobalt-rich crust exploration contract area in the Western Pacific by the 51st expedition of the China Oceanic Survey in 2018 (149°48′ E and 16°51′ N, about 1600 m water depth, and the cutting net sampling method). The cobalt-rich crust contract area is located in the Magellan Seamount Group in the Western Pacific Ocean (Figure 1a). The Magellan Seamount Group has a complex structure in the sea, and a series of transformation faults are developed in the ocean floor, which are NW trending. The main volcanoes in the Magellan Seamount Group are speculated to be of hot spot origin. After formation, the seamounts migrated to the northwest continuously; therefore, the seamounts in the southeast direction are younger than in the northwest direction. During the migration of seamounts, many periods of magmatic activity occurred due to passing several hot spots [24,25]. The overall colour of the samples was light greyish brown to greyish brown, and they were all regular spherules with a diameter of more than 5 cm (Figure 1b,c). A smooth surface, which indicates a slower growth rate, is usually hydrogenic (Figure 1b).

## 3. Analytical Methods

### 3.1. Optical Microscopy

Polymetallic nodules were processed by 1/2 and 1/4 splitting methods, the former for viewing the cross section of nodules and the latter for making thin sheets for optical microscopy. Thin sheets of polymetallic nodules were observed by a ZEISS scope A1 optical microscope (Zeiss, Oberkochen, Germany) in the Laboratory of Marine Energy and Sustainable Development, School of Marine Science, Sun Yat-sen University. The mineral morphology in the core and microstructure and their changes in the shell of polymetallic nodules were observed.

### 3.2. X-ray Diffraction Analysis

The mineral composition of polymetallic nodules was analysed by XRD. In the laboratory of the School of Marine Science, at Sun Yat-sen University, powder was collected from the core and shell of the polymetallic nodule samples and treated using the 1/2 splitting method with an electric grinding machine and measured by D/Max Rapid ⅱ. Subsequently, the powder obtained at 10 points in different shells of polymetallic nodules were tested and analysed in the Test Center of Sun Yat-sen University using an Empyrean Nano multifunctional X-ray diffractometer. All powder samples were scanned from 5° to 70° (2θ). The data processing of the samples was carried out by using Jade software supplemented by PDF2 database analysis.

### 3.3. X-ray Fluorescence Spectroscopy

The study of geochemical characteristics of polymetallic nodules, from macroscopic to microscopic, observes the distribution characteristics of constituent elements. Microarea X-ray fluorescence surface scanning was performed to study the overall macroscopic distribution of geochemical elements at Guangzhou Tuoyan Testing Technology Co., Ltd. The samples were thin slices of polymetallic nodules treated by the 1/4 splitting method and tested by the M4 Plus Microzone X-ray fluorescence analyser produced by Brock Company (Kowloon City, Hong Kong).

### 3.4. SEM and EDS

The observation and distribution of component elements of polymetallic nodules under a microscope were studied. Preliminary SEM observations were conducted in Guangzhou Tuoyan Testing Technology Co., Ltd, Guangzhou, China, which used the TESCAN MIRA3 (TESCAN, Brno, Czech Republic). In the Test Center of Sun Yat-sen University, the mineral morphology of the polymetallic core samples was further observed from the microscopic perspective. A Gemini 500 high resolution thermal field emission scanning electron microscope (Zeiss, Oberkochen, Germany) was used. Flat-q was used in the appropriate areas of the core and shell to conduct surface and line scanning test analysis of the elemental geochemical characteristics of the samples. The sample was run on the tuberculosis sample light plate, made by Guangzhou Tuoyan Testing Technology Co., Ltd. Before observation, the light plate is treated with carbon spraying or gold plating to increase electrical conductivity.

## 4. Results

### 4.1. Micromorphology and Microstructure

**Figure 2:**
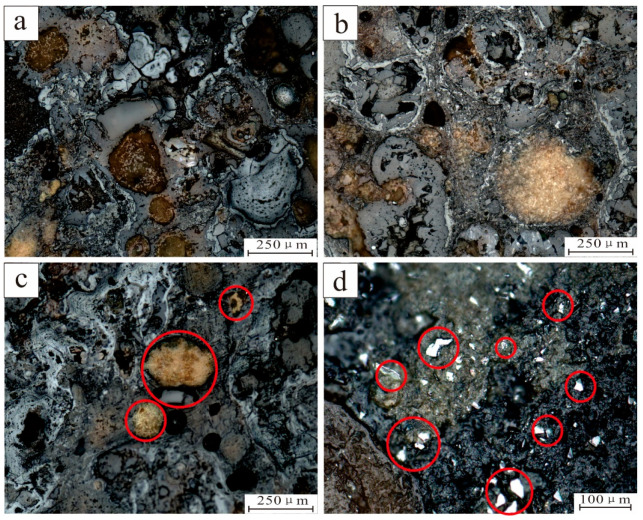
Mineral distribution and morphology of polymetallic nodules. (**a**,**b**) Distribution of terrigenous clast minerals in polymetallic nodules; (**c**) Feldspar particles (the red circle) from the core of polymetallic nodules; (**d**) Quartz mineral particles (the red circle) at the core of polymetallic nodules.

**Figure 3:**
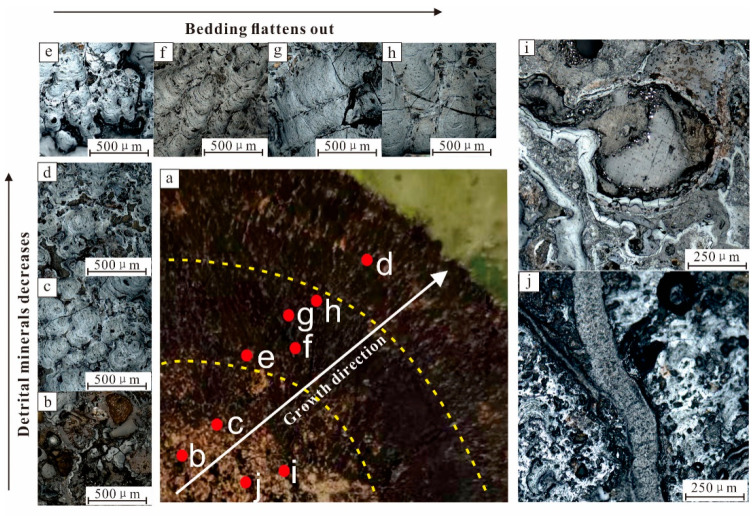
Microstructural characteristics of polymetallic nodule crust. (**a**) growth direction of the polymetallic nodule; (**b**) mottled structure; (**c**) oncentric spherical structure; (**d**) laminated structure; (**e**) columnar structure; (**f**) stronmatolitic structure; (**g**) massive structure; (**h**) petal-shaped structure; (**i**) vein-like structure; (**j**) filling-like structure. The yellow dotted line is the boundary between the loose and dense layers. (**b**,**c**) are in the loose core; d is in the loose shell; (**e**–**h**) are in dense shell; (**i**,**j**) are the secondary structure in the loose core.

The initial observations differed between the nucleus and shell under an optical microscope (Figure 2 and Figure 3). The core had a high content of detrital minerals with different shapes (Figure 2a,b). Quartz particles were larger and well rounded (Figure 2c), while fine feldspar particles usually form mineral aggregates with ferro-manganese phase minerals with low crystallinity (Figure 2d). The crust was composed of ferro-manganese minerals with a low degree of crystallization, which made it difficult to observe single particles using optical microscope methods, since only the microscopic structure characteristics can be seen (Figure 2).

Microstructures in the shell show certain regularities. Shells from the core to the edge present a detrital mineral content that initially decreased, and then increased, showing a loose to tight change. The microscopic structure was characterized by a loose shell near the nuclear division, mainly at the spot of mixed structure. The concentric spherical structure, with a constant growth shell, evolves into the laminated structure, column structure, and tuberculosis in the central dense shell. The structure was primarily laminar, and some massive structures can be seen. After the dense crust, it evolves into loose crust again and develops speckled and petal-like structures. Secondary structures, such as vein-like structures and filling structures, develop in some areas of the nodules, which indicate that the polymetallic nodules have undergone some late transformation (Figure 3).

### 4.2. Mineralogy and Micromorphology

**Figure 4:**
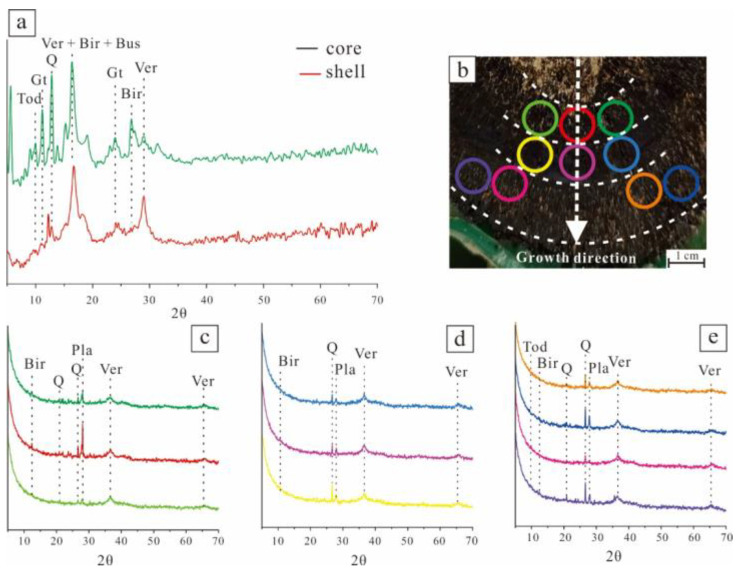
X-ray diffraction of the core and shell of polymetallic nodules. (**a**) XRD of the core and shell; (**b**) sampling position of XRD in polymetallic nodules; (**c**) XRD of the loose layer (inner); (**d**) XRD of the dense layer; (**e**) XRD of the loose layer (outer). Gt—goethite, Q—quartz, Ver—vermiculite, Bir—birnessite, Bus—bustamite, Pla—Plagioclase, Tod-todorokite.

Polymetallic nodules of different origins generally show different mineral composition characteristics. Through the XRD analysis of the core and shell of polymetallic nodules, the mineral compositions of nodule samples were preliminarily studied, and the different shells were further tested based on observations under a microscope (Figure 4a,b).

Obvious differences are shown in the diffraction peaks of the core and shell (Figure 4a). The diffraction peaks of the core are numerous and mixed, with obvious detrital mineral peaks, such as quartz; the shell has few but clear diffraction peaks. The most obvious diffraction peak was seen in Vernadite. The diffraction peak of detrital minerals such as quartz in the core was obvious and stronger than that in the shell. The shell was mainly composed of manganese minerals such as Vernadite, birnessite and todorokite. Among manganese minerals, hydromanganite was dominant.

Further determination of different shell layers of polymetallic nodules was conducted (Figure 4c–e). The XRD diffraction curves of different layers differ mainly in the types and quantities of diffraction peaks of the manganese phase minerals and the intensity of clastic mineral peaks. The XRD diffraction peaks of the manganese phase minerals in the middle of the shell of polymetallic nodules are the lowest, and only the hydromanganite diffraction peaks are obvious. In the area closest to the core of the shell there are diffraction peaks of sodium manganese hydrate; in the outer part of the shell, there are diffraction peaks of barium magnesium manganese and sodium manganese hydrate. The crust nearest to the core and the outermost crust have higher clastic mineral peaks.

**Figure 5:**
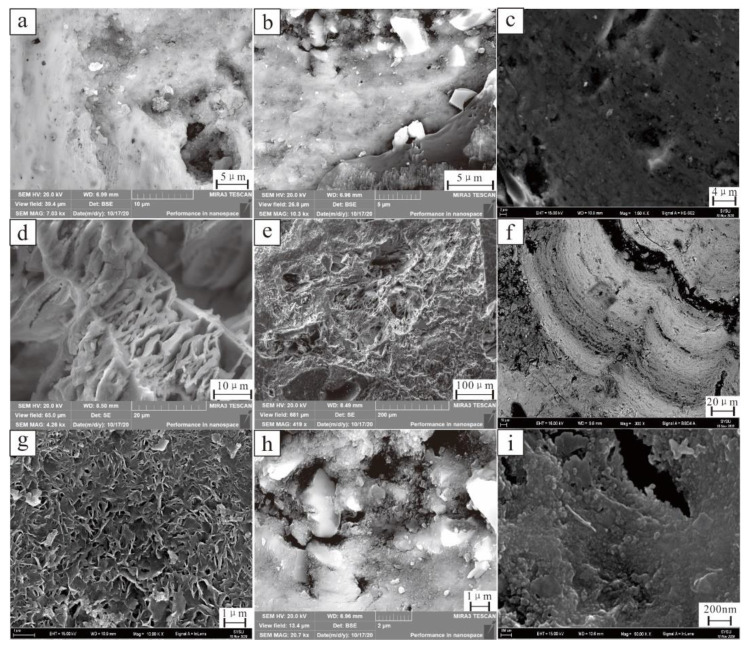
SEM images of the core of polymetallic nodules. (**a**) polymetallic nodule core; (**b**) quartz grains; (**c**) feldspar; (**d**) altered feldspar; (**e**) clay minerals; (**f**) iron-manganese phase minerals; (**g**) clay minerals in a dense shell; (**h**) growing quartz grains in a shell; (**i**) nanomineral particles.

The morphology of polymetallic nodules was observed by SEM (Figure 5). The core and shell show obvious differences. The pores in the core are well developed (Figure 5a), and the quartz particles are mainly granular and have different shapes under the microscope (Figure 5b). Feldspar particles are plate-like with pores in the middle; thickness of a single layer is approximately 1 μm (Figure 5c). Part of the feldspar was altered (Figure 5d). Microscopically, except for detrital minerals, feldspar are generally iron-manganese minerals with a low degree of crystallization, and it is difficult to observe single mineral grains (Figure 5f).

The composition of the polymetallic nodule shells is relatively uniform, closely arranged as a whole, and the pores are rare or not developed (Figure 5g). At normal magnification, the resulting microstructure was observed as the shell grew (Figure 5h). When the SEM magnification was increased to 50 KX, single mineral particles were observed (Figure 5i). The particle shape was regular and basically spherical, with a size of approximately 10 nm, which was typical of manganese oxide minerals and nanomineral particles.

### 4.3. Geochemical Element Distribution Results

Based on the macroscopic morphology, the microscopic structure and the mineral microscopic morphology of nodule samples by optical microscopy and SEM, XRF and EDS were used to test the geochemical macroscopic and microscopic distribution of polymetallic nodules.

**Figure 6:**
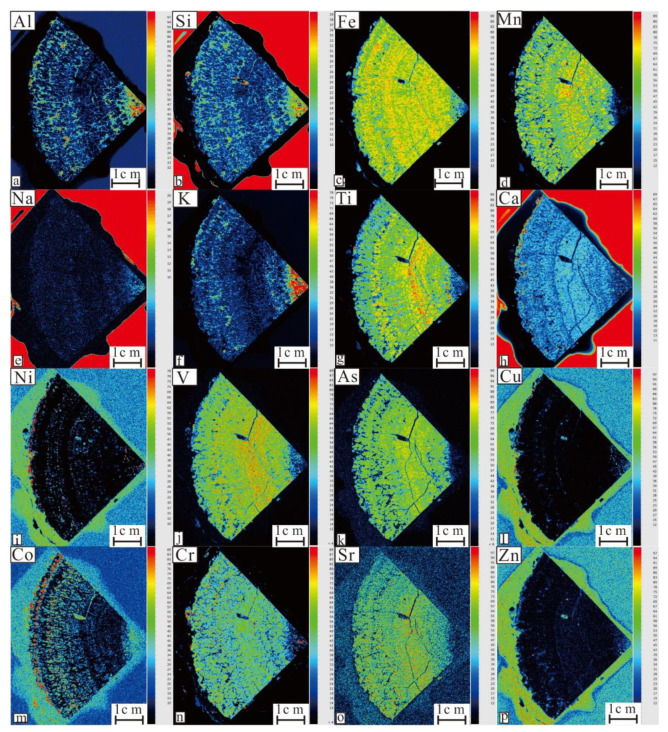
XRF scan results of multi-elements in the polymetallic nodule profile. (**a**) Al element scan result; (**b**) Si element scan result; (**c**) Fe element scan result; (**d**) Mn element scan result; (**e**) Na element scan result; (**f**) K element scan result; (**g**) Ti element scan result; (**h**) Ca element scan result; (**i**) Ni element scan result; (**j**) V element scan result; (**k**) As element scan result; (**l**) Cu element scan result; (**m**) Co element scan result; (**n**) Cr element scan result; (**o**) Sr element scan result; (**p**) Zn element scan result; (The red indicates the most element content and blue indicates the least element content).

The XRF results (Figure 6) show that Al, Si, Na, and K, the constituent elements of aluminosilicate minerals, are mainly distributed in the core of the nodules; elements mainly derived from seawater, such as Sr and As, are mainly distributed in the nodule shell. Other elements show no obvious difference between the core and shell. Fe, Mn, Ti, and Ca, with high element contents were distributed in cloddy, while Ni, Co, and Mg were relatively low in content and distributed in spots. V and Cr are observed both in cloddy and spots. Cu and Zn, which are usually abundant in nodules, were very low in these samples, and their distribution was not obvious.

**Figure 7:**
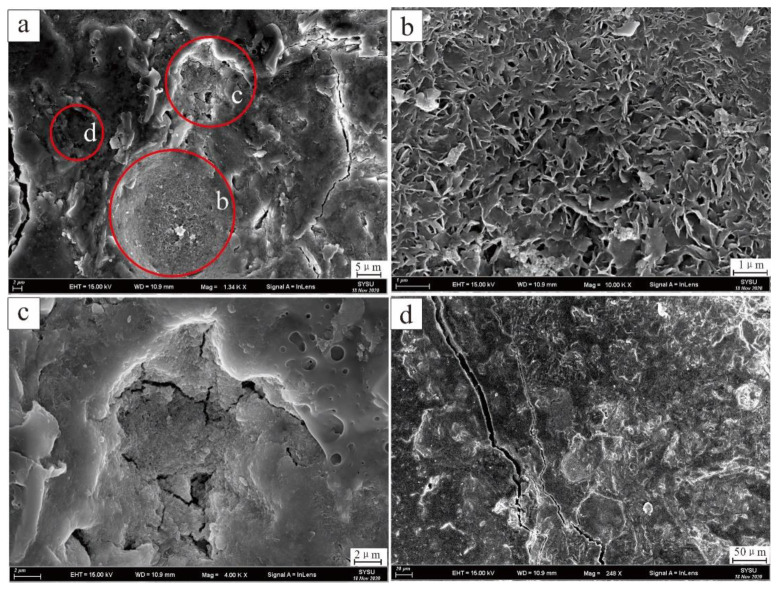
Division map of polymetallic nodules EDS scan area. (**a**) total area; (**b**) region 1; (**c**) region 2; (**d**) region 3.

**Figure 8:**
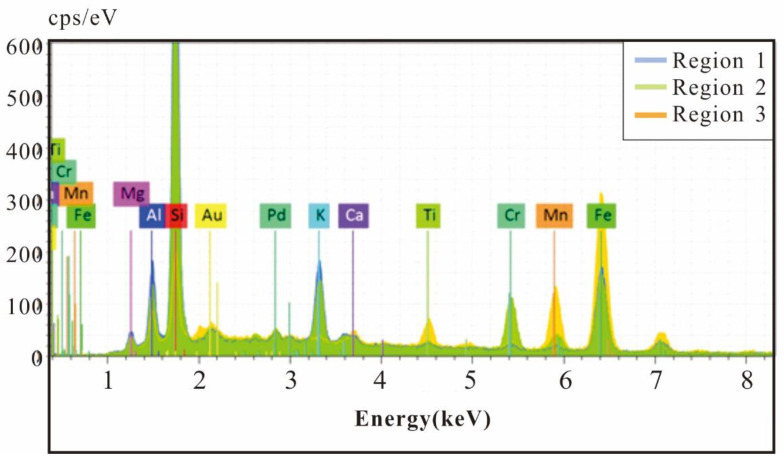
EDS area scan results of the core in polymetallic nodules.

According to the SEM image results of polymetallic nodules, EDS surface scanning was carried out in the core region of nodules to test and analyse the geochemical element characteristics. Three areas with typical features were selected for the study (Figure 7), and the scanning results are shown in Figure 8 and Table 1.

Region one (Table 1 and Figure 7b and Figure 8) is the largest selected region, and the scanning results show that this region is mainly composed of Si, Fe, and Al. Combined with the mineral morphology characteristics, the area described is mainly flake clay minerals with a small amount of quartz particles. In region two (Table 1 and Figure 7c and Figure 8) the smallest area was selected, and the scanning result curve of the region was roughly similar to that of region one; however, the Cr peak was the highest among the three regions. SEM images in area three (Table 1 and Figure 7d and Figure 8) are quite different from those in region one and region two, in which mineral particles are closely arranged and pores are rare or not developed. The EDS surface scanning results of region three are completely different from those of region one and region two. The main elements in region three were Fe and Mn, while the Si signal peak was significantly lower than that in region one and region two.

**Figure 9:**
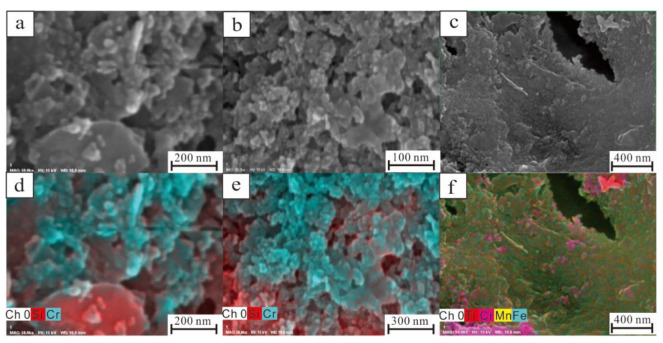
Nanomineral particles in polymetallic nodules. (**a**) Nanomineral particles in region 2; (**b**) nanomineral particles in region 3; (**c**) nanomineral particles in the shell; (**d**–**f**) corresponding distributions of Cr, Si, Mn, and Fe elements.

Under normal magnification it was difficult to observe single mineral particles in regions two and three, but when the magnification reaches 50 KX, the distribution of spherical nanomineral particles in the regions could be observed. The EDS surface scanning results in this region show that the main element composition of irregular flake mineral particles is Si, while Cr is mainly concentrated in the spherical nanometre mineral particle region (Figure 9a,b,d,e).

The EDS surface scanning results show that the content of Si is significantly reduced, and Mn is significantly increased in the shell of polymetallic nodules compared to the core; however, Cr is mainly distributed in spherical nanomineral particles (Figure 9c,f), which is the same as the core. Ti is concentrated in irregular particles in EDS, which is speculated to be biological debris (Figure 9c,f).

## 5. Discussion

### 5.1. Relationship between Nanomineral Particles and the Formation of Polymetallic Nodules

Widely distributed nanomineral particles were observed in polymetallic nodules. By observing the morphology of nanomineral particles and combining the geochemical and mineralogical data of polymetallic nodule samples, the special properties of nanomineral particles in polymetallic nodules and the solid–liquid interface process were preliminarily understood based on previous research results [15,25].

During the formation and growth of single polymetallic nodules the environment changes, and the factors controlling the aggregation of nanomineral particles in the environment change accordingly, which affect the aggregation behaviour of nanomineral particles and the growth of polymetallic nodules. On a global scale, each ocean has different environmental conditions, and the aggregate growth of nanomineral particles in water is affected to different degrees, which affects the growth and preservation of nodules and changes the distribution of polymetallic nodules in the global ocean to a certain extent.

Mineral-water interfacial reactions exist widely in nature and are extremely important to all geological processes because they involve the cycling of geochemical elements. In recent years, with the development of science and technology, a new possibility is provided to understand the reaction mechanism between minerals and the water interface by directly observing the reactions in the nanometre range of the mineral surface.

When the magnification reaches 50 KX, nanomineral particles in polymetallic nodules can be observed (Figure 7 and Figure 9). When combined with EDS, it can be observed that the distribution of Cr elements has obvious selectivity, mainly distributed in spherical nanomineral particles (Figure 9). Due to its own characteristics, manganese oxide minerals have a strong adsorption capacity for many heavy metal elements and can oxidize and change the valence of heavy metal elements to change their morphology and toxicity. The only known oxidant of Cr in nature is manganese oxide, so it plays an important role in the migration, cycling and distribution of Cr [26,27].

**Figure 10:**
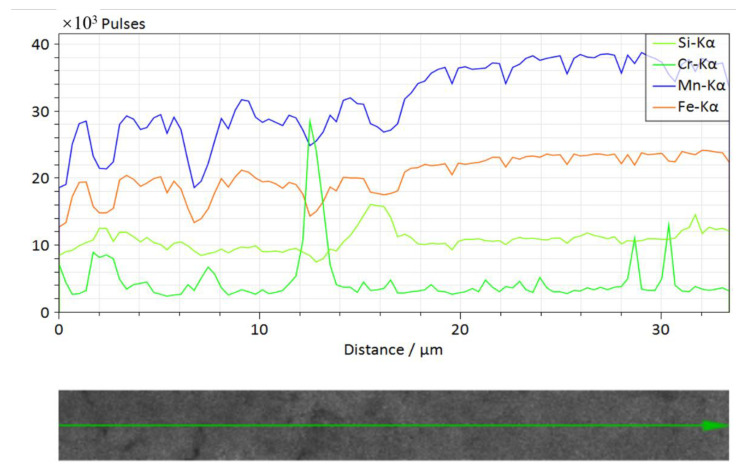
EDS line scanning results in the shells of polymetallic nodules. (The direction of the arrow represents the direction of polymetallic nodule growth).

XRF (Figure 6) and EDS (Figure 8 and Figure 9) results show that Cr elements were abundant in polymetallic nodules, showing a patch-like distribution. Further line scanning of EDS (Figure 10) show that the trend changes of Fe and Mn elements were relatively consistent, and the Fe/Mn ratio between them maintained a stable size, which indicate that the growth environment of nodules at this stage was relatively stable. However, the peak value of Cr appeared in the low value area of Fe and Mn, the two elements of Fe and Mn were significantly negatively correlated with Cr, and the scanning area where nanomineral particles were exposed. The Cr element in the nanomineral particles had more obvious enrichment than others with large particle sizes.

### 5.2. Growth Stage of Polymetallic Nodules

According to the microstructural and mineralogical characteristics of polymetallic nodules in the western Pacific Ocean, the growth of polymetallic nodules mainly occurs in three stages. In growth stages, different solid–liquid interface processes of nanomineral particles are dominant; however, they play an important role in the growth of polymetallic nodules [28,29].

**Figure 11:**
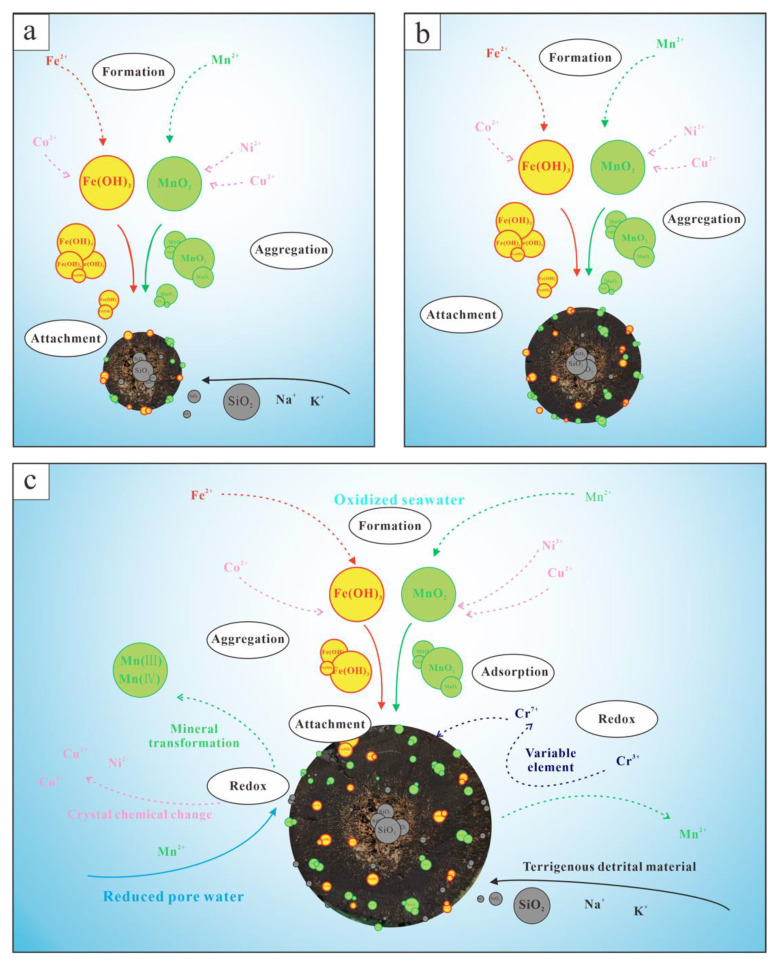
Schematic diagram of the growth stages of polymetallic nodules in the Western Pacific Ocean. (**a**) the first stage; (**b**) the second stage; (**c**) the third stage.

In the first growth stage of polymetallic nodules, the nucleus of polymetallic nodules is formed, and a loose shell near the core is formed (Figure 11a). Due to the large input of terrigenous material and strong hydrodynamic conditions in the initial growth stage, the shell formed is loose and porous, and most has a patchy and mixed structure. In the mineral composition the content of detrital minerals is high, consisting mainly of quartz and feldspar, and the morphology is different. The manganese phase minerals are mainly Vernadite, which is a typical mineral of hydrogenesis; there is a small amount of Birnessite. At this stage, the Fe and Mn in seawater, which are the main components of polymetallic nodules, are oxidized in oxidized seawater to form Vernadite and goethite, respectively, which are typical nanominerals. After the formation of nanomineral particles, homogeneous aggregation easily occurs, attaches to the nucleus formed earlier, and grows to form the innermost shell.

The second growth stage of polymetallic nodules is the stable growth stage, in which a dense layer in the middle of the polymetallic nodule shell is formed. In this stage, the hydrodynamic conditions were weak, and the influence of terrigenous material input was small. Lamellar structures were often developed, and some were massive structures. The mineral composition is mainly Vernadite, and the content of detrital minerals is very low. At this stage, the interfacial process of nanomineral particles formed by Fe and Mn is still dominated by aggregation growth (Figure 11b).

The third stage is the last growth stage of polymetallic nodules, which forms the porous layer at the edge of the polymetallic nodules and experiences the most abundant changes (Figure 11c). The hydrodynamic conditions became stronger, the input amount of terrigenous material and the petrogenic element content increased, and the microscopic structure evolved into a speckle structure. The addition of reducing pore water changes the geochemical environment in which polymetallic nodules grow. The Vernadite was transformed by diagenesis to form Todorokite, and the Mn (IV) in the mineral structure is reduced to form Mn (II), resulting in the change of Cu, Co and Ni in the structure. Due to redox reactions, the geochemistry and crystal geochemistry of the variable valence elements present in the nodules change in different valence states, which affect the element contents in the nodules. The nanomineral particles also experience the most abundant interface process. Redox changes lead to the internal structure of the particles and the adsorption behaviour and performance in this stage, due to the selective adsorption of Cr, especially with increasing time. The reduction environment of nanomineral particles will eventually dissolve, leading to the disappearance of polymetallic nodules.

## 6. Conclusions

(1) A large number of nanomineral particles, mainly composed of Fe, Mn and Cr, develop in the cores and shells of polymetallic nodules in the western Pacific Ocean. Some elements in the nanomineral particles have more obvious enrichment than others with large particle sizes and can affect the formation and growth of polymetallic nodules.
(2) The agglomeration and adsorption of nanomineral particles play an important role in the growth process of nodules. The growth process of polymetallic nodules can be divided into three stages. In the first stage, terrigenous detritus nucleates and begins to adsorb nanomineral particles composed of Fe, Mn and other elements to form the first shell. In the second stage, a dense shell layer is formed under stable conditions, and the redox conditions of the nodules change and are modified by diagenesis in the third stage.

## Figures and Tables

**Table 1:** Normalized mass concentration (The value is the percentage of content).

Regions	Mg	Al	Si	K	Ca	Ti	Cr	Mn	Fe	Pd	Au
1	2.90	8.21	37.84	7.66	0.82	0.64	1.12	1.51	35.24	1.63	2.41
2	2.16	6.16	30.63	5.90	0.60	0.88	12.99	3.75	32.67	1.60	2.66
3	2.54	3.31	5.86		1.0	3.58	1.11	18.23	61.43	0.96	2.34

Note: The sample was sprayed with gold before using EDS surface scanning, resulting in high content of Pd and Au.

## Data Availability

Not applicable.
